# *Lactococcus lactis* NCC 2287 Alleviates Food Allergic Manifestations in Sensitized Mice by Reducing IL-13 Expression Specifically in the Ileum

**DOI:** 10.1155/2012/485750

**Published:** 2011-09-22

**Authors:** Adrian W. Zuercher, Marietta Weiss, Sébastien Holvoet, Mireille Moser, Hélène Moussu, Laurence van Overtvelt, Stéphane Horiot, Philippe Moingeon, Sophie Nutten, Guénolée Prioult, Anurag Singh, Annick Mercenier

**Affiliations:** ^1^Allergy Group, Department of Nutrition and Health, Nestle Research Center, Vers-chez-les-Blanc, 1000 Lausanne 26, Switzerland; ^2^CSL Behring AG, Wankdorfstraße 10, 3010 Bern 22, Switzerland; ^3^Applied Mathematics Group, Department of Bio-Analytical Sciences, Nestle Research Center, Vers-chez-les-Blanc, 1000 Lausanne 26, Switzerland; ^4^Stallergènes SA, Research and Development, 6 rue Alexis de Tocqueville, 92183 Antony, France

## Abstract

*Objective*. Utilizing a food allergy murine model, we have investigated the intrinsic antiallergic potential of the *Lactococcus lactis* NCC 2287 strain. *Methods*. BALB/c mice were sensitized at weekly intervals with ovalbumin (OVA) plus cholera toxin (CT) by the oral route for 7 weeks. In this model, an oral challenge with a high dose of OVA at the end of the sensitization period leads to clinical symptoms. *Lactococcus lactis* NCC 2287 was given to mice via the drinking water during sensitization (prevention phase) or after sensitization (management phase). *Results*. *Lactococcus lactis* NCC 2287 administration to sensitized mice strikingly reduced allergic manifestations in the management phase upon challenge, when compared to control mice. No preventive effect was observed with the strain. *Lactococcus lactis* NCC 2287 significantly decreased relative expression levels of the Th-2 cytokine, IL-13, and associated chemokines CCL11 (eotaxin-1) and CCL17 (TARC) in the ileum. No effect was observed in the jejunum. *Conclusion/Significance*. These results taken together designate *Lactococcus lactis* NCC 2287 as a candidate probiotic strain appropriate in the management of allergic symptoms.

## 1. Introduction

The prevalence of allergic diseases has been increasing dramatically in the past decades [1, 2]. Allergic sensitization starts in early childhood mainly to common food allergens encountered in everyday food products such as cow's milk, eggs, and wheat. Subsequent exposure to the allergen involves an intricate interplay of cellular components of the adaptive immune system in which CD4+ T cells are activated to secrete cytokines such as IL-4, IL-5, and IL-13 [3, 4]. The development of allergic manifestations can be altered via two approaches, one in which sensitization to new allergens is prevented, thereby inhibiting the development of the Th-2 conditioning [5]. We refer to this approach as “prevention” in the context of our study. Once sensitization to the allergen has occurred, subsequent exposure can trigger allergic symptoms; the effective management of these allergic manifestations then becomes the primary goal. We have named this approach “management” in relation to our study. 

Probiotics are defined by the WHO as “living micro-organisms that when administered in adequate amounts confer a health benefit to the host” [6]. Among potential health promoting attributes, the capacity of probiotic strains to modulate the host immune system, either by direct signaling or by modulating the intestinal microbiota, is currently an area of intense research. The beneficial role of probiotics, especially *Lactobacillus* and *Bifidobacterium* strains in atopic diseases, has been investigated with increasing interest over the past few years with both animal studies [7, 8] and human clinical trials [9–12]. These studies have yielded conflicting results that in part reflect the diversity of clinical settings studied as well as the different probiotic strains that have been investigated. The importance of intervening at the appropriate time window in relation to allergies, that is, either by preventing sensitization or in management of allergic symptoms, has remained under investigated. 

Based on extensive *in vitro* immune profiling of different candidate probiotic strains using both murine and human cell-based assays, we selected for the current study a lactic acid bacterial strain *Lactococcus lactis* (*L. lactis*) NCC 2287. We sought to evaluate the benefits of intervening at the two phases, that is, prevention and management, in a murine model of food allergy via *L. lactis* NCC 2287. *Lactococcus* strains have been used to deliver therapeutic molecules [13] but have rarely been individually studied in disease models for their probiotic effects. We report that while no preventive effect with the strain was observed, *L. lactis* NCC 2287 administration to sensitized mice strikingly reduced allergic scores induced upon oral challenge in the management phase when compared to control mice. In addition, we investigated the different mechanisms via which *L. lactis* NCC 2287 may exert its therapeutic effect. *L. lactis* NCC 2287 administration during the management phase leads to a decrease in IL-13 production from restimulated mesenteric lymph node (MLN) cells along with a significant decrease in the relative expression levels of IL-13 and Th-2 associated chemokines CCL11 (eotaxin-1) and CCL17 (TARC) in the ileum but not in the jejunum.

## 2. Materials and Methods

### 2.1. Reagents and Bacterial Biomass

*Lactococcus lactis *(*L. lactis*) strain NCC 2287 is a dairy starter strain from the Nestlé culture collection (NCC) that was deposited at Collection Nationale de Cultures de Microorganismes at Institut Pasteur, Paris, France (CNCM I-4154). Bacterial biomass was produced by culture of NCC 2287 under standard conditions. Growth curve was determined for the strain, and according to this, bacterial cells were harvested by centrifugation 3 h after entering in the stationary phase. The biomass was washed 2x in cold PBS and frozen in PBS 20% glycerol at −80°C.

### 2.2. OVA Food Allergy Murine Model

**Figure 1:**
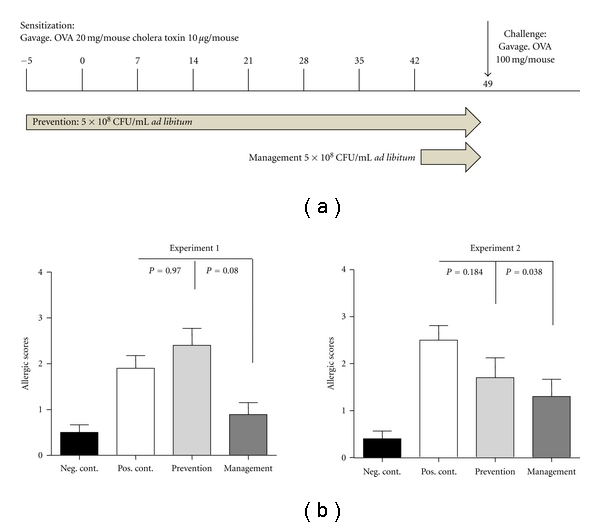
*L. lactis* NCC 2287 alleviates allergic symptoms in sensitized mice. *L. lactis* NCC 2287 (5 × 10^8^ CFU/mL) was given *ad libitum* to mice (*n* = 10, *n* = 5 in Neg. control) orally via drinking water (a). Administration was either before the first sensitization and given throughout the experiment (prevention phase) or in the one week after the last sensitization (management phase). After challenge, mice treated with *L. lactis* NCC 2287 in the management phase (dark grey bar graph) developed significantly reduced clinical scores than sensitized, untreated animals in the positive control group (white bar graph). Mice consuming *L. lactis* NCC 2287 during the prevention phase of the experiment (grey bar graph) did not exhibit reduced symptoms. An exploratory experiment 1 (left panel) and a confirmatory experiment 2 (right panel) are shown. 3 experiments were performed in total (b).

All animal studies were approved by a Nestec internal Ethics Committee and the Service Vétérinaire of the Canton of Vaud, Switzerland (Authorization no. 1970). This model has been described in detail before [14]. Briefly, six-week old conventional female BALB/c mice (Harlan Laboratories, France) were sensitized (*n* = 10 per group; negative control *n* = 5) orally via gavage at weekly intervals by 20 mg of ovalbumin (OVA) (Fluka, Buchs, Switzerland) and 10 *μ*g/mouse of Cholera toxin (CT) (List Biologicals, purchased from LuBioscience, Lucerne, Switzerland) for 7 consecutive weeks. Animals were challenged orally via gavage with 100 mg of OVA one week after the last sensitization (Figure 1(a)). Starting 30 minutes after challenge, mice were individually observed for 30 min. Clinical symptoms were recorded and quantified as follows (allergic score): 0: no symptoms, less than 4 episodes of scratching; 1: 4–10 episodes of scratching around the nose and head, no diarrhoea; 2: more than 10 episodes of scratching or bristled fur and immobility or soft stool; 3: diarrhoea or laboured respiration or cyanosis; 4: diarrhoea in combination with immobility after prodding, bristled fur, laboured respiration or cyanosis; 5: anaphylaxis. Mice demonstrating a symptom severity of ≥4 were sacrificed immediately. Four hours after challenge, mice were sacrificed after isoflurane anaesthesia and terminal bleeding. Blood and the last centimetre of ileum and jejunum were taken and frozen in liquid nitrogen. *L. lactis* strain NCC 2287 (5 × 10^8^ CFU/mL in drinking water) was administered at different phases of the experiment and its effect was compared to the positive (OVA + CT) control groups. To evaluate the efficacy during the prevention phase, we administered the probiotic starting 5 days before the first oral sensitization. Administration was then continued during the entire experimental period. To assess the effect of administering the probiotic in sensitized mice, *L. lactis* NCC 2287 was provided in drinking water starting after the last sensitization up to the challenge with OVA for a total duration of 8 days (management phase).

### 2.3. Quantification of Serum Levels of Mouse Mast-Cell Protease 1 (MMCP-1)

MMCP-1 was analyzed in mouse serum by ELISA, purchased from Moredun Scientific (Penicuik, Scotland) according to the manufacturer's instructions. The MMCP-1 concentration was obtained by converting OD values in pg/mL using a polynomial standard curve.

### 2.4. Quantification of Serum Levels of OVA-Specific IgE, IgG1, and IgG2a

OVA-specific immunoglobulin-E (IgE), immunoglobulin-G1 (IgG1), and immunoglobulin-G2 (IgG2a) concentrations were measured by ELISA as described previously [14, 15]. For IgE measurement, plates (NUNC Maxisorp; VWR, Nyon, Switzerland) were coated overnight at 4°C with rat antimouse IgE (2 *μ*g/mL; BD Pharmingen, Allschwil, Switzerland). After washing, wells were blocked with PBS-1% BSA for 1 h at RT. Serially diluted sera and standard (monoclonal mouse anti-OVA; ABD Serotec, Düsseldorf, Germany) were incubated for 2 h at 37°C. Then, biotinylated-OVA (1 *μ*g/mL) was added to the plate for 1 h at 37°C, followed by incubation with HRP-labeled streptavidin (1 : 1000; KPL; Socochim, Lausanne, Switzerland) for 30 min at 37°C. Plates were developed with tetramethylbenzidine (TMB) substrate (KPL). The reaction was stopped with 1 M HCl (Merck, Darmstadt, Germany). Optical densities were measured at 450 nm. Concentrations were calculated by converting OD values in pg/mL using a polynomial standard curve. For IgG1 and IgG2a, microtiter plates were coated with OVA (Sigma, Buchs, Switzerland) (100 *μ*g/mL) overnight at 4°C. Wells were washed with PBS 0.05% Tween (Biorad, Reinach, Switzerland) and then blocked with PBS-1% BSA for 1 h at room temperature. Serially diluted standard (monoclonal mouse anti-OVA IgG1 and anti-OVA IgG2a from Antibody Shop; LucernaChem, Lucerne, Switzerland) and serum samples were incubated for 2 h at 37°C, followed by incubation for 2 h with a HRP-labelled goat antimouse IgG1 or IgG2a antibody (1 : 5000; Southern Biotech; Bioconcept, Allschwil, Switzerland). Plates were then developed, read, and analyzed.

### 2.5. Isolation and Culture of MLN Cells

Mesenteric lymph nodes (MLN) were homogenized with a syringe plunger in a cell strainer (BD Falcon; Milian, Meyrin, Switzerland). Cells were centrifuged and washed 2x in RPMI medium (Sigma) complemented with 10% fetal bovine serum (FBS; Bioconcept, Paris, France), 1% L-glutamine (Sigma), 1% Penicillin/Streptomycin (Sigma), 0.1% Gentamycin (Sigma), 50 *μ*M *β*-mercaptoethanol (Sigma). Cells (3 × 10^5^ cells/well) were cultured in 96 well flat bottom plates (Corning, Milian, Meyrin, Switzerland) in the absence or presence of OVA (1 mg/mL). After 72 hrs of culture, plates (including supernatant and cells) were frozen at −20°C.

### 2.6. Quantification of Cytokines in Culture Supernatant Fluid

Mouse IL-4, IL-5, and IL-10 were measured using the mouse Th-1/Th-2 multiplex kit (Meso Scale Discovery, Gaithersburg, Md, USA) according to the manufacturer's instructions. IL-13 was measured using a Mouse IL-13 (DY413E) ELISA kit from R&D Systems (Abington, England).

### 2.7. Quantitative Gene Expression Levels by Low-Density Array (LDA)

Total ribonucleic acids (RNAs) from ileum and jejunum were extracted according to the manufacturer's protocol using the SV Total RNA Isolation System kit (Promega, Dübendorf, Switzerland). RNA was quantified with quant-IT Ribogreen Reagent kit purchased from Promega according to the manufacturer's protocol. Reverse transcription was performed on 1 *μ*g of total RNA by using the Multiscribe Reverse Transcriptase kit (Applied Biosystems, Foster City, Calif, USA). Total RNA was mixed with 50 *μ*M of random hexamers, 0.5 mM of dNTPs, 20 U of RNase inhibitor (Applied Biosystems), 62.5 U of Multiscribe reverse transcriptase, 1X RT buffer, and 5.5 mM of MgCl_2_ in a final volume of 50 *μ*L. Reverse transcription was performed on a T3 thermocycler (Biometra, Göttingen, Germany) with the following cycle program: 10 min at 25°C, 30 min at 48°C, 5 min at 95°C to finish at 4°C. Low-density arrays were designed online on the Applied Biosystems website. The load and the run were performed according to the manufacturer's protocol on a quantitative ABI-Prism 7900HT. The quantification was normalized with the mean of 3 house-keeping genes: *β*-actin, GAPDH, and HPRT. The Ct value for each gene was corrected by the Ct mean of these three house-keeping genes. Based on the cycle threshold (Ct) values obtained, a relative and normalized mRNA expression was determined for each gene using the ΔCt. The results were calculated as a relative expression using the formula 2^−ΔCt^ × *K*, where *K* is a 10^6^ factor. Fold increase results expression was normalized to expression levels in the negative control group.

### 2.8. Statistical Analyses

The software R 2.2.1 was used for the analyses. Clinical scores were evaluated using the Kruskal-Wallis tests, followed by Wilcoxon test. All other outcomes were treated with Kruskal-Wallis followed by Wilcoxon test. Corrections were applied following the Bonferroni-Sidak procedure. Statistical test to compute *P* values are calculated for median ± SEMedian values. Results were considered as significant with a *P* value ≤ 0.05.

## 3. Results

### 3.1. *L. lactis* NCC 2287 Oral Administration Is Effective in the Management of Food Allergy Symptoms but has no Effect on the Prevention of Sensitization

The *in vivo* effect of *L. lactis *NCC 2287 was tested in a murine model of food allergy in the prevention of allergic sensitization as well as in the management of allergic symptoms in sensitized mice (Figure 1(a)). For this purpose, BALB/c mice were sensitized to OVA via the oral route and challenged as described in Section 2. *L. lactis *NCC 2287 was given to mice via drinking water (5 × 10^8^ CFU/mL; *ad libitum*) during the prevention phase (5 days before the first sensitization until the end of the experiment) or in the last week of the experiment following the last sensitization (day 43–49; management phase).  Figure 1(b) illustrates the clinical symptoms observed in different groups of mice in two representative experiments. After challenge, animals in the positive control group developed statistically significant clinical scores of food allergy compared to the negative control group (clinical score of 1.9 ± 0.3 versus 0.5 ± 0.17; *P* < 0.008 in exploratory experiment 1 and clinical score of 2.5 ± 0.31 versus 0.4 ± 0.16; *P* < 0.001 in confirmatory experiment 2). Sensitized mice treated with *L. lactis *NCC 2287 in the management phase developed less severe clinical symptoms than the positive control group (0.9 ± 0.26; *P* = 0.08 in exploratory experiment 1 and 1.3 ± 0.37; *P* = 0.038 in confirmatory experiment 2). However, mice consuming *L. lactis *NCC 2287 strain during the prevention phase of the experiment did not exhibit significantly reduced symptoms upon OVA challenge (2.4 ± 0.37; *P* = 0.97 in exploratory experiment 1 and 1.7 ± 0.42; *P* = 0.18 in confirmatory experiment 2). These findings suggest that *L. lactis* NCC 2287 strain acts more likely during the management phase following challenge and not during the prevention phase.

### 3.2. *L. lactis* NCC 2287 Administration Does Not Influence Levels of OVA-Specific IgE, IgG1, or Mouse Mast-Cell Protease- 1 (MMCP-1) in Sensitized Mice

**Figure 2:**
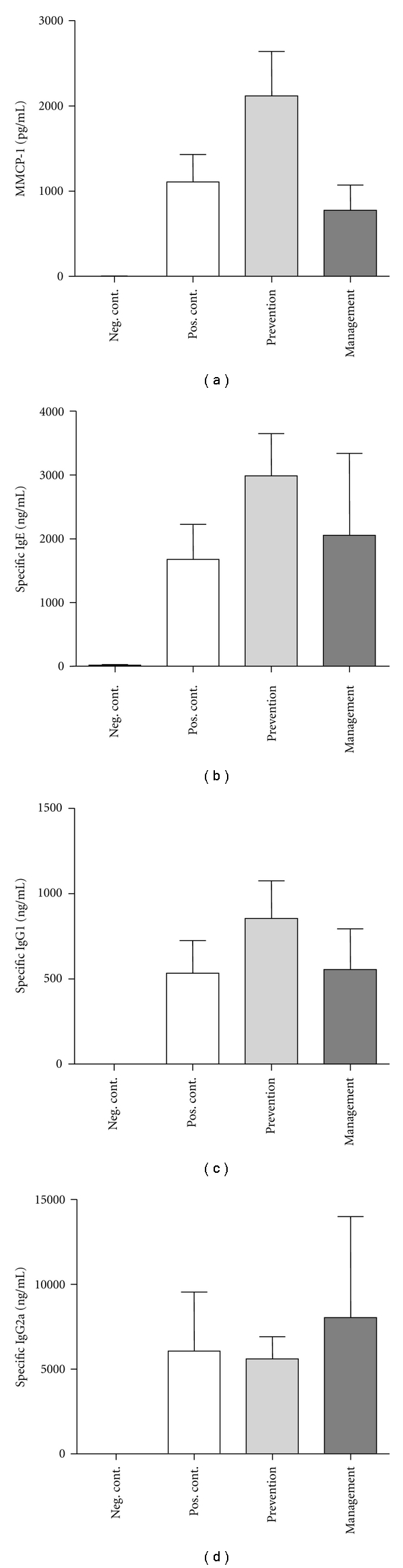
MMCP-1 (a), OVA-specific IgE (b), IgG1 (c), and IgG2a (d) levels in the serum 4 hours after challenge. Results from the confirmatory experiment are shown. OVA-specific levels of IgE, IgG1, and IgG2a were increased significantly in positive control mice compared to the negative control group. Administration of NCC 2287 both in the prevention and management phases did not significantly reduce levels of MMCP-1 and OVA-specific IgE, IgG1, and IgG2a. No effect of *L. lactis *NCC 2287 on mast cells and immunoglobulins.

In order to determine which immunological parameters contributed to the observed beneficial effect of *L. lactis* NCC 2287 on clinical scores, we investigated the impact on serum levels of mouse mast-cell protease 1 (MMCP-1) and OVA-specific antibodies (IgE, IgG1, and IgG2a). MMCP-1 levels were increased significantly in the positive control group of animals (1107 ± 322 pg/mL) when compared to negative control, nonsensitized mice (3.8 ± 2.2 pg/mL; *P* ≤ 0.001). Administration of *L. lactis *NCC 2287 did not statistically decrease MMCP-1 in either the preventive (2113 ± 521 pg/mL; *P* = 0.17) or the management (773 ± 298 pg/mL; *P* = 0.48) phases of the model (Figure 2(a)). OVA-specific IgE (Figure 2(b)), IgG1 (Figure 2(c)), and IgG2a (Figure 2(d)) levels increased significantly in the positive control group (1674 ± 551 pg/mL for IgE, 535 ± 192 pg/mL for IgG1 and 6058 + 3474 pg/mL for IgG2a) when compared to the negative control group (19 ± 11 pg/mL for IgE and below detection level for IgG1 and IgG2a; *P* ≤ 0.001). However, the administration of *L. lactis* NCC 2287 did not significantly impact the levels of the immunoglobulin subtypes, neither in prevention (2986 ± 661 pg/mL for IgE, 857 ± 221 pg/mL for IgG1 and 5603 + 1306 pg/mL for IgG2a; *P* ≥ 0.1) or in symptom management (2054 ± 1281 pg/mL for IgE, 558 ± 238 pg/mL for IgG1 and 8045 ± 5951 pg/mL for IgG2a; *P* ≥ 0.3) phases of the model (Figures 2(b)–2(d)). These data suggest that *L. lactis* NCC 2287 is unlikely to lead to a reduction in allergic scores via its effect on two pathways commonly involved in the allergic cascade, namely, mast cell activation and humoral immunity.

### 3.3. Impact of Administering *L. lactis* NCC 2287 during Management Phase on IL-13 Production by MLN Cells

**Figure 3:**
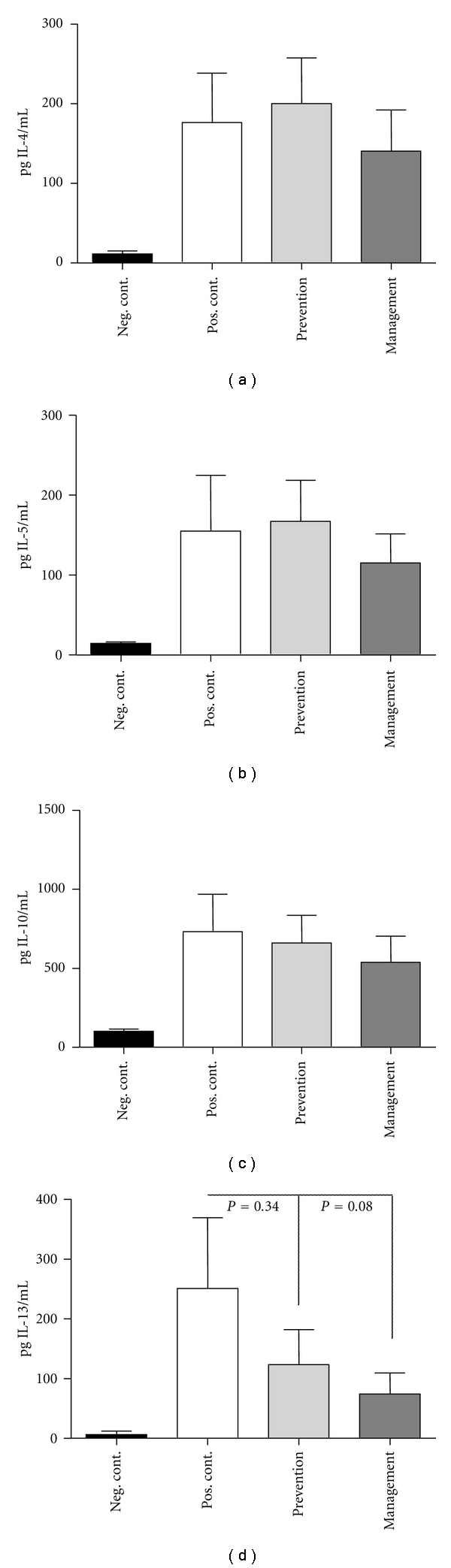
Decreased IL-13 production from restimulated MLN cells in mice administered *L. lactis *NCC 2287 in the management phase. IL-4 (a), IL-5 (b), IL-10 (c), and IL-13 (d) levels in the supernatants of MLN cells restimulated *ex vivo* with OVA. Positive control group secreted increased levels of Th-2 cytokines. MLN cells from mice administered *L. lactis* NCC 2287 during the prevention phase exhibited similar levels of Th-2 cytokines as the positive control group. MLN cells of mice administered *L. lactis* NCC 2287 during the management phase produced decreased levels of IL-13 cytokine levels but not IL-4, IL-5, or IL-10.

To further identify potential mechanisms via which *L. lactis* NCC 2287 exerted its beneficial effect, we next evaluated the *ex vivo* cytokine profile of antigen-restimulated lymphocytes isolated from the MLN. As shown in Figure 3, MLN cells from the negative control group secreted low levels of Th-2 cytokines (IL-4, IL-5, IL-10, and IL-13). In comparison, MLN cells from the positive control group secreted increased levels of IL-4 (Figure 3(a)), IL-5 (Figure 3(b)), IL-10 (Figure 3(c)), and IL-13 (Figure 3(d)). MLN cells from mice administered *L. lactis* NCC 2287 during the prevention phase exhibited similar levels of Th-2 cytokines as the positive control group. Of interest were the MLN cells of mice given *L. lactis* NCC 2287 during the management phase that demonstrated a trend to decreased IL-13 cytokine production (*P* = 0.08) but not IL-4, IL-5, and IL-10 levels compared to the positive control.

### 3.4. *L. lactis* NCC 2287 Administration during Management Phase Reduced Relative Gene Expression Levels of the Th-2 Cytokine IL-13 in the Ileum but not in the Jejunum of Sensitized Mice

**Figure 4:**
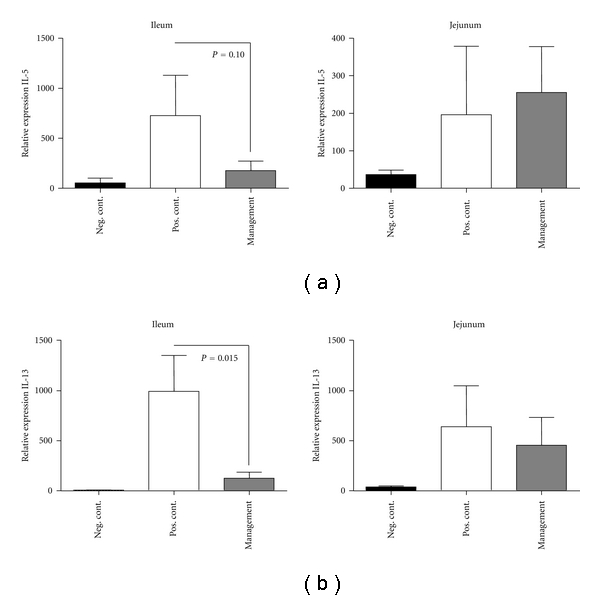
*L. lactis *NCC 2287 downregulates IL-13 expression in the ileum but not in the jejunum of sensitized mice. Relative gene expression of IL-5 (a) and IL-13 (b) in samples from ileum and jejunum analyzed by low-density arrays. Sensitization followed by challenge led to upregulation of IL-5 (*P* = 0.08) and IL-13 (*P* = 0.001) mRNA in the positive control group compared to the negative control in the ileum. Administration of NCC 2287 in the management phase led to a marked downregulation of IL-13 expression in the ileum (*P* = 0.015) but not in the jejunum.

To correlate the results obtained *ex vivo* with OVA restimulation on IL-13, we investigated gene expression levels of IL-13, locally in the gastrointestinal tract. In addition, we also examined the expression of the IL-5 encoding gene. For this purpose, we isolated tissue samples from ileum and jejunum and examined relative gene expression levels by RT-PCR. IL-5 gene expression showed an increased trend in the ileum of positive control mice when compared to the negative control group (726 ± 403 in positive control versus 55 ± 47 in negative control; *P* < 0.08). The administration of *L. lactis* NCC 2287 in sensitized mice resulted in lower relative expression levels of IL-5 in the ileum (177 ± 95; *P* = 0.1 when compared to the positive control group); however, this effect was not statistically significant. There was no impact on IL-5 gene expression levels in the jejunum (Figure 4(a)). IL-13 gene expression levels on the other hand were remarkably increased in the ileum (992.6 ± 360.2 in positive control versus 7.3 ± 1.2 in negative control; *P* ≤ 0.01). Interestingly, the administration of *L. lactis* NCC 2287 strongly mitigated the increase in the relative gene expression level for IL-13 (124 ± 62; *P* = 0.015 when compared to the positive control group) in the ileum of sensitized mice following challenge. There was no effect on IL-13 relative gene expression in the jejunum of mice administered* L. lactis* NCC 2287 during the management phase (Figure 4(b)). 

### 3.5. Decreased Th-2 Chemokines CCL11 (Eotaxin-1) and CCL17 (TARC) Gene Expression Levels in the Ileum Following *L. lactis* NCC 2287 Administration during the Management Phase

**Figure 5:**
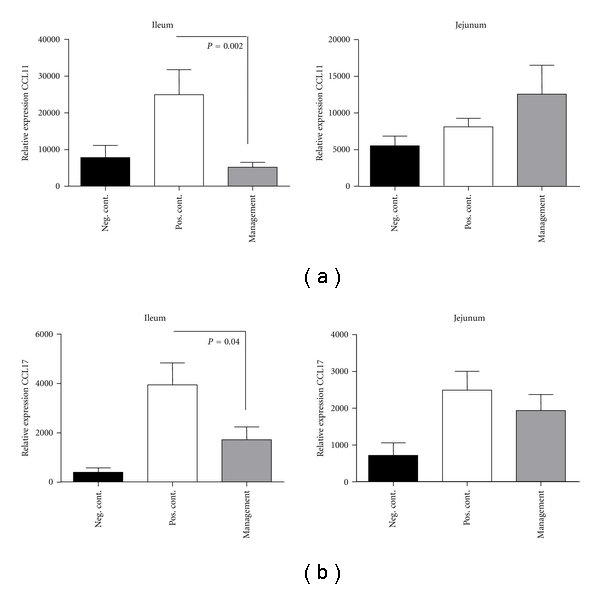
*L. lactis* NCC 2287 administration to sensitized mice downregulates Th-2 chemokines CCL11 (eotaxin-1) and CCL17 (TARC) in the ileum but not in the jejunum. Relative gene expression for CCL11 (a) and CCL17 (b) in samples from ileum and jejunum analyzed by low-density arrays. Sensitization followed by challenge led to upregulation of CCL11 (*P* = 0.006) and IL-13 (*P* = 0.001) mRNA in the positive control group compared to the negative control in the ileum. Administration of NCC 2287 in the management phase led to a marked downregulation of both CCL11 (*P* = 0.002) and CCL17 expression (*P* = 0.04) in the ileum but not in the jejunum.

Chemokines associated with allergic disorders and upregulated in the presence of elevated levels of IL-13 such as CCL11 (eotaxin-1) and CCL17 (TARC) were analyzed. Sensitization and subsequent challenge resulted in a significant increase in the relative gene expression levels of the chemokine CCL11 (eotaxin-1) in the ileum of positive control mice in comparison to the negative control group (24904 ± 6797 versus 7809 ± 3286 in negative control; *P* = 0.006; Figure 5(a)). A similar statistically significant up-regulation was also observed on the relative gene expression levels of CCL17 in the positive control group (3943 ± 888 versus 400 ± 177 in negative control; *P* = 0.001; Figure 5(b)). The administration of *L. lactis* NCC 2287 in the management phase significantly reduced the relative gene expression levels of both CCL11 (5172 ± 1341; *P* = 0.002 versus positive control group) and CCL17 (1716 ± 521; *P* = 0.04 versus positive control group). Similarly, as observed for IL-13, no effect on CCL11 or CCL17 gene expression levels was observed in other intestinal sites such as jejunum.

## 4. Discussion

The current study explored the potential efficacy and mechanism of action of *L. lactis *NCC 2287 against the development of food allergy manifestations. Specifically, we delineated the efficacy of this strain in two critical phases of a food allergy mouse model, namely, by intervention during the sensitization phase (prevention phase) and in the symptomatic phase by intervention in sensitized mice shortly before challenge with the objective to manage allergic symptoms (management phase). When administered *in vivo, L. lactis* NCC 2287 significantly reduced food allergic symptoms in sensitized mice. Interestingly, the administration of the strain during sensitization was without any effect (Figure 1(b)). This underlines that certain probiotic strains may be “specialized” in modulating different phases of the allergic response. This is one of the most important findings of the current study and underlines that the efficacy of probiotic interventions is strongly dependent on the specific strain(s) used, of the clinical indication for which the strain is applied and the appropriate timing of the intervention. 

The reduction of symptoms following *L. lactis* NCC 2287 administration in the management phase was not associated in the studied animal model with a reduction in levels of OVA-specific antibodies associated with a Th-2 phenotype, that is, IgE, IgG1, and IgG2a (Figures 2(b)–2(d)). These results are not completely surprising, since the levels of antigen specific immunoglobulins build up over time with repeated sensitizations, leading to a long-term persistence of memory B cells. Previous studies have also reported similar results upon the administration of probiotics in different allergy models [16, 17]. As we had previously observed an association between decreased MMCP-1 levels, a marker for mast cell activation and reduced allergic scores with a nutritional intervention in the same animal model [15], we also evaluated this parameter in the present study; the strain did not impact the levels of MMCP-1. It is then likely that mechanistically the antiallergic effect of this strain is not mediated by direct inhibition of mast-cell degranulation. Indeed, confirmatory *in vitro *experiments with IgE-sensitized RBL cells also did not support an inhibitory effect of *L. lactis* NCC 2287 on mediator release (data not shown). 

In our model, the reduction in clinical scores in the management phase was paralleled by a decrease in the secretion of IL-13 by OVA restimulated MLN cells (Figure 3(d)) but not in IL-4, IL-5, or IL-10 (Figures 3(a)–3(c)). IL-13 is a vital Th-2 cytokine that dominates the chronic phase of allergic sensitization and is primarily involved in the recruitment of eosinophils to sites of allergic inflammation and their subsequent activation and survival at these inflammatory sites [18, 19]. IL-13 is secreted by a variety of immune cells such as Th-2 cells, mast cells, dendritic cells, and eosinophils [20, 21]. IL-13 has been closely linked to the pathogenesis of a food allergic disorder, eosinophilic esophagitis both in animal studies and in clinical samples obtained from individuals with allergic disorder [22, 23]. IL-13 is also closely related to another well-studied Th-2 cytokine, IL-4 with respect to structure and function as both cytokines share a common receptor (IL-4R*α*) [24, 25]. While relative gene expression levels of IL-4 were undetectable, we examined expression patterns of IL-5 and IL-13 following *L. lactis* NCC 2287 administration (Figure 4). There was nearly a 10-fold reduction in the relative gene expression of IL-13 in the ileum. This dramatic reduction was not observed elsewhere in the gastrointestinal tract, that is, jejunum (Figure 4). These results are also in line with our previous observations *in vitro* with *L. lactis* NCC 2287 in assays including monocyte derived DCs-resting CD4+ T cell cocultures and Th-2 skewed PBMC stimulation. *L. lactis* NCC 2287 was selected for its potential Th1/Treg immunomodulatory profile and its ability to inhibit the levels of Th2 cytokines in particular IL-5 and IL-13 (unpublished). These are intriguing results and suggest a mechanism whereby *L. lactis* NCC 2287 exerts its probiotic effect at a specific site of localized inflammation. 

Supporting the local reduction of IL-13, we observed a statistically significant decrease in relative gene expression levels of Th-2 associated chemokines mainly CCL11 and CCL17 (Figures 5(a) and 5(b)). These chemokines and particularly CCL11 (eotaxin-1), are critically linked downstream to IL-13 signaling [26–28]. CCL17 (TARC) has previously been reported to be associated with allergic disorders [29] and potentially altered by probiotics in *in vitro* systems mimicking allergic pathogenesis [30]. The reduction of CCL11 and CCL17 expression was also observed only in the ileum further providing support to this site being an “active” site of inflammation in a gastrointestinal food allergy immune response as well as a site of action of *L. lactis* NCC 2287. 

Multiple biological pathways exist that can contribute to the regulation of an aberrant immune response as is the case with food allergies. We have identified in this study one of the many mechanisms that could be responsible for the initiation of food allergic symptoms, mainly increased levels of IL-13 locally in the ileum and its downregulation upon administration of a probiotic *L. lactis* NCC 2287 in the management phase. The fact that the probiotic effect was observed for the strain only in sensitized mice suggests that* L. lactis* NCC 2287 acts specifically at sites of allergic inflammation. The survival rate and physiology of *L. lactis* in the digestive tract have previously been studied. In these observations, the *L. lactis* strain MG 1363 was reported to have a higher survival rate in the ileum in rodents [31]. These studies raise the possibility that in the mouse model used in this study, a similar preferential localization of the strain can happen in the ileum upon oral administration. Given such a scenario, the strain would then indeed have the maximum impact during the challenge phase of the model in the ileum. It would be interesting to study the effect of *L. lactis* NCC 2287 in either different models of allergy (skin and respiratory) and to compare the effect to other *L. lactis* strains. This approach of studying a bacterial strain for its probiotic effect in different phases of a disease model is the “way forward” for a more rational and practical approach to the selection of different strains for a particular health benefit.
